# Exercise Prevents Enhanced Postoperative Neuroinflammation and Cognitive Decline and Rectifies the Gut Microbiome in a Rat Model of Metabolic Syndrome

**DOI:** 10.3389/fimmu.2017.01768

**Published:** 2017-12-11

**Authors:** Xiaomei Feng, Yosuke Uchida, Lauren Koch, Steve Britton, Jun Hu, David Lutrin, Mervyn Maze

**Affiliations:** ^1^Center for Cerebrovascular Research, Department of Anesthesia and Perioperative Care, University of California San Francisco, San Francisco, CA, United States; ^2^Department of Anesthesiology, University of Michigan Medical School, Ann Arbor, MI, United States; ^3^Department of Molecular and Integrative Physiology, University of Michigan Medical School, Ann Arbor, MI, United States; ^4^Department of Anesthesia, Tongling People’s Hospital, Tongling, China

**Keywords:** preoperative exercise, postoperative cognitive decline, postoperative neuroinflammation, microbiome, metabolic syndrome

## Abstract

**Introduction:**

Postoperative cognitive decline (PCD) can affect in excess of 10% of surgical patients and can be considerably higher with risk factors including advanced age, perioperative infection, and metabolic conditions such as obesity and insulin resistance. To define underlying pathophysiologic processes, we used animal models including a rat model of metabolic syndrome generated by breeding for a trait of low aerobic exercise tolerance. After 35 generations, the low capacity runner (LCR) rats differ 10-fold in their aerobic exercise capacity from high capacity runner (HCR) rats. The LCR rats respond to surgical procedure with an abnormal phenotype consisting of exaggerated and persistent PCD and failure to resolve neuroinflammation. We determined whether preoperative exercise can rectify the abnormal surgical phenotype.

**Materials and methods:**

Following institutional approval of the protocol each of male LCR and male HCR rats were randomly assigned to four groups and subjected to isoflurane anesthesia and tibia fracture with internal fixation (surgery) or anesthesia alone (sham surgery) and to a preoperative exercise regimen that involved walking for 10 km on a treadmill over 6 weeks (exercise) or being placed on a stationary treadmill (no exercise). Feces were collected before and after exercise for assessment of gut microbiome. Three days following surgery or sham surgery the rats were tested for ability to recall a contextual aversive stimulus in a trace fear conditioning paradigm. Thereafter some rats were euthanized and the hippocampus harvested for analysis of inflammatory mediators. At 3 months, the remainder of the rats were tested for memory recall by the probe test in a Morris Water Maze.

**Results:**

Postoperatively, LCR rats exhibited exaggerated cognitive decline both at 3 days and at 3 months that was prevented by preoperative exercise. Similarly, LCR rats had excessive postoperative neuroinflammation that was normalized by preoperative exercise. Diversity of the gut microbiome in the LCR rats improved after exercise.

**Discussion:**

Preoperative exercise eliminated the metabolic syndrome risk for the abnormal surgical phenotype and was associated with a more diverse gut microbiome. Prehabilitation with exercise should be considered as a possible intervention to prevent exaggerated and persistent PCD in high-risk settings.

## Introduction

Postoperative cognitive decline (PCD) is a devastating complication with long-lasting consequences especially in elderly surgical patients. PCD encompasses both the DSM V-defined Postoperative Delirium as well as the imprecise entity of postoperative cognitive dysfuction whose existence has been challenged (1). Devising strategies to limit the occurrence and/or severity of PCD will require a thorough understanding of the underlying pathophysiological mechanisms.

By interpreting data acquired from preclinical studies of PCD, we propose that aseptic peripheral trauma engages the innate immune response through cellular disruption and release of cytosol-located high molecular group box 1 protein (HMGB1) at the sites of tissue damage (2). HMGB1 initiates synthesis and release of proinflammatory cytokines through activation of nuclear factor kappa-light-chain-enhancer of activated B cells (NF-κB) in circulating bone marrow-derived monocytes (BM-DMs) (2). HMGB1 also upregulates monocyte chemoattractant protein-1 (MCP-1) in the central nervous system (2). Peripheral inflammation disrupts the blood–brain barrier, allowing CCR2-expressing BM-DMs to enter the hippocampus, attracted by its cognate ligand, MCP-1 (3). Together with the CNS-residing immunocompetent microglia that become activated (4), the BM-DMs generate a neuroinflammatory response that interferes with long-term potentiation, a form of synaptic plasticity required for learning and memory (5). In most settings, both inflammation and cognitive decline are short-lived as neural (6) and humoral (7) mechanisms promptly resolve the inflammation.

Patients with metabolic syndrome are particularly prone to develop PCD (8, 9). To further explore the reasons for this enhanced risk, we have used a validated animal model in which low capacity runner (LCR) rats, bred for their limited aerobic exercise capacity, exhibit each of visceral obesity, hypertension, hyperlipidemia, and insulin resistance, the cardinal features of the metabolic syndrome (10). After aseptic trauma, the LCR rats develop an exaggerated and more persistent form of cognitive decline (11) that is associated with a failure of resolution of inflammation (11).

Aerobic exercise training attenuates inflammation by altering signaling in innate immune cells (12–16). Exercise has also been shown to limit inflammation in type 2 diabetic patients (17). Therefore, we sought to determine whether exercise rectifies the neuroinflammatory response to surgical trauma in LCR rats and whether the exaggerated and persistent PCD can be mitigated. As an altered gut microbiome may be a driver for persistent inflammation (18) we also determined the effect of exercise on the diversity of the microbiome in LCR rats.

## Materials and Methods

### Animals

**Figure 1:**
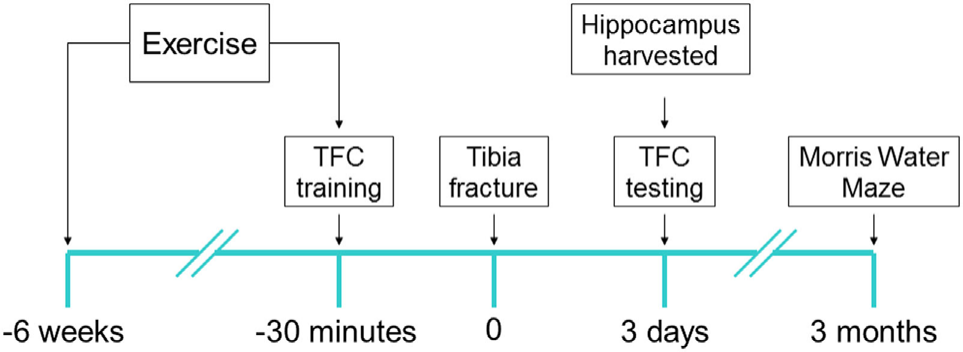
Experimental design: each of low capacity runner and high capacity runner rats were randomly assigned into groups to receive interventions of exercise/no exercise and surgery/sham surgery. Exercise-assigned rats exercised on a treadmill for 6 weeks prior to surgery/sham surgery. 30 min prior to tibia fracture (surgery) under general anesthesia or sham surgery, the training session for trace fear conditioning (TFC) was performed and context testing was performed 72 h later (6–8/group). Immediately after TFC context testing rats were sacrificed and the brain was harvested. Separate cohorts (5–7/group) were tested for cognition 3 months after surgery using Morris Water Maze probe test.

All experimental procedures involving animals were approved by Institutional Animal Care and Use Committee of University of California, San Francisco (protocol no.: AN167062), and conformed to National Institute of Health guidelines (Figure 1). All animals were housed (two rats per cage) in saw dust-lined cages in an air-conditioned environment with 12-h light/dark cycles and were fed standard rodent chow and water *ad libitum*. LCR rats and high capacity runner (HCR) rats were developed by Koch and Britton using the 35th generation (19). Rats were randomly allocated to surgery or sham group before any procedure was undertaken and researchers were blinded to the group assignment during assessments, prior to the analysis phase.

### Surgery

Under general anesthesia with 2.1% isoflurane in 0.30 FiO_2_, rats underwent an open tibial fracture of the left hind paw with intramedullary fixation under aseptic surgical conditions as previously described (11, 20). Briefly, under sterile conditions a 20 G pin was inserted in the intramedullary canal of the left tibia, and osteotomy was performed after the periosteum was stripped. During the surgical procedure that lasted approximately 10 min, temperature was monitored and maintained at 37°C with the aid of warming pads (Harvard Apparatus, Holliston, MA, USA). Buprenorphine (0.1 mg/kg) was given subcutaneously to provide analgesia after the induction of anesthesia and before skin incision. The sham rats were exposed to anesthesia and analgesia as above.

### Exercise

Rats, both LCR as well as HCR, were exposed to a 6-week treadmill exercise protocol before surgery similar to Britton and Koch’s exercise protocol with some modifications (10). During the first week of the protocol, rats were acclimated to treadmill exercise for gradually increasing duration each day beginning with 1 min at a velocity of 10 m/min and by day 5 rats exercised for 5 min. From week 2, rats exercised 5 days a week over 6 weeks with an identical exercise protocol for the same week gradually achieving 31.5 min at a speed of 20 m/min. Exercise animals ceased their training 48 h prior to further experimental procedures. The control (non-exercise) rats were placed on a non-moving treadmill daily for an equivalent duration to the time that the exercise rats spent on the moving treadmill.

### Behavioral Studies

#### Trace Fear Conditioning

Trace fear conditioning was used to assess memory in rodents as previously described (11, 20). In brief, the training chamber is connected to a shock delivery system (Med Associates, St. Albans, VT, USA). During the training, rats were allowed to explore this context for 3 min after which they were presented with a conditional stimulus, an auditory cue (75–80 dB, 5 kHz,) for 20 s. Twenty seconds after termination of the auditory tone, the unconditional stimulus, a 2-s foot shock (0.8 mAmp) was administered. Rats were removed from the training chamber after an additional 30 s. Surgery was performed within 30 min after training. Three days later, rats were placed back into the same chamber (“context”) in which it was trained but with neither tone nor shock. The memory of the learned fear was assessed by freezing behavior each 5 s during the 5 min observation time. The percentage of the observation period that the animal adopted freezing behavior was calculated using the formula 100 × *f/n*, where *f* is the number of freezing events per rat and *n* is the total number of observations per rat.

#### Morris Water Maze

Three months after surgery, rats were investigated in the Morris Water Maze as previously reported and now briefly described (11).

#### Cued Trials

The cued trial includes three training sessions with a visible platform by placing a visible yellow flag on top of the platform; the platform was relocated between each session.

#### Visuospatial Reference Memory

The platform is hidden in this trial. The rat was released from the assigned location while facing the wall of the tank. Six fixed locations were randomly selected to generate one long, one medium, and one short swim every session. Equal numbers of rats were randomly allocated to one of the four quadrants of platform locations in order to minimize any bias related to platform location. 90 s were set as a cutoff value. The rats were trained until they could locate the hidden platform in less than 15 s, on average, within a session.

#### Probe Trials

Immediately after the last training session (in which rats could locate the platform ≤ 15 s), the 60-s probe trial, reflected by memory retention for the hidden platform location, was performed with the platform removed from the tank. Time spent in the target quadrant, where the platform formerly resided, was recorded as the dwelling time. Swimming speed, and time spent in the target quadrant were analyzed using an EthoVision video tracking system (Noldus Instruments, Wageningen, Holland).

### Inflammation

#### Hippocampal IL-6 Protein Expression

Three days after surgery, rats were sacrificed under isoflurane anesthesia. The hippocampus was harvested and stored at −80°C for further analysis. The hippocampus from one side was homogenized in cell lysis buffer (Cell Signaling Technology), mixed with phenylmethanesulfonyl fluoride (Cell Signaling Technology) and protease inhibitor (Thermo Fisher Scientific). Protein concentration was assayed with Pierce BCA Protein Assay kit (Thermo Fisher Scientific). Interleukin IL-6 was measured using a commercially available ELISA kit following the manufacturer’s instructions (R&D Systems).

#### Hippocampal Inflammatory Mediators mRNA Expression by Quantitative Polymerase Chain Reaction (q-PCR)

The hippocampus from the other side was harvested at 3 days after surgery was placed in RNAlater^TM^ solution (Qiagen). RNA extraction, RNA-to-cDNA reverse transcription, and q-PCR were performed as previously described (20). Relative gene expression for IL-6, HMGB-1, MCP-1, Integrin alpha-X (Itgax), Netrin-1 (Ntn1), Arginase 1(Arg1), and Mannose receptor C type 2 (Mrc2), were calculated using the comparative threshold cycle ΔΔCt and housekeeping gene β-actin for normalization of gene expression. The mRNA levels for target genes are expressed as fold increases relative to LCR + sham + exercise group.

### Gut Microbiome

Feces were collected from rats prior to exercise and after exercise. Samples were provided to Microbiome Core at University of California, San Francisco for DNA extraction, PCR amplification of the V4 hypervariable region of the 16sRNA gene, and DNA sequencing on the Illumina MiSeq. DNA was extracted from the samples using the MoBio PowerSoil DNA Isolation Kit according to the manufacturer’s recommendations. For each sample, the PCR was amplified in triplicate using primer pairs that (i) targeted the V4 hypervariable region of the16srRNA gene, (ii) contained a unique barcode sequence to enable demultiplexing of pooled samples, and (iii) contained an adapter sequence that enables the amplicon to bind to the MiSeq flowcell. Amplicons were pooled in equimolar concentrations and sequenced on the Illumina MiSeq. Paired sequencing reads were quality filtered and demultiplexed using the QIIME software package before being assembled and processed further. Briefly, assembled sequencing read pairs were binned into operational taxonomic units (OTUs) using a 97% similarity to the green genes database; reads that neither clustered to the green genes database nor were chimeric were removed from subsequent analyses. Sample read numbers were rarefied to the read number of the lowest sample after processing (88,048) resulting in a rarefied OTU table. From this OTU table downstream analysis was performed, and alpha and beta diversity indices were calculated.

### Statistical Analysis

All data in this study were analyzed using Prism 6.0 (GraphPad Software, San Diego, CA, USA) and were expressed as mean ± SD. Statistical comparison was performed by a one-way ANOVA followed by Newman–Keuls multiple comparison test for *post hoc* analysis. Significance was set at *P* < 0.05.

## Results

### Preoperative Exercise and Body Weight

**Figure 2:**
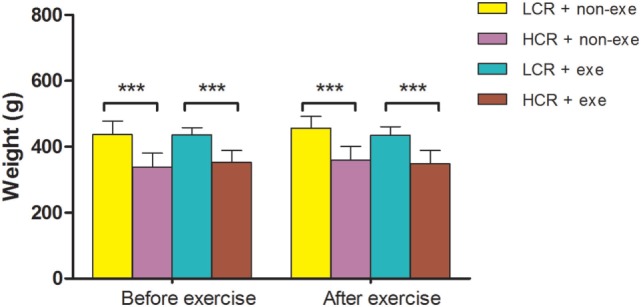
Exercise and body weight: LCR and HCR rats were randomly assigned into four groups (*n* = 12–14) and trained with and without exercise. Exercise was performed 5 days per week for 6 weeks. Body weight (g) was recorded before exercise and immediately after exercise and is expressed as means ± SD, and analyzed by one-way ANOVA and Newman–Keuls *post hoc* analysis. ***P < 0.001 for comparisons shown.

As diet was not restricted exercise alone did not significantly alter body weight in the groups (*n* = 12–14/group) (Figure 2). The relative difference in body weight between the LCR and HCR rats that was present before exercise was sustained after exercise.

### Preoperative Exercise Attenuated Exaggerated Acute Cognitive Decline in Metabolic Syndrome Rats

**Figure 3:**
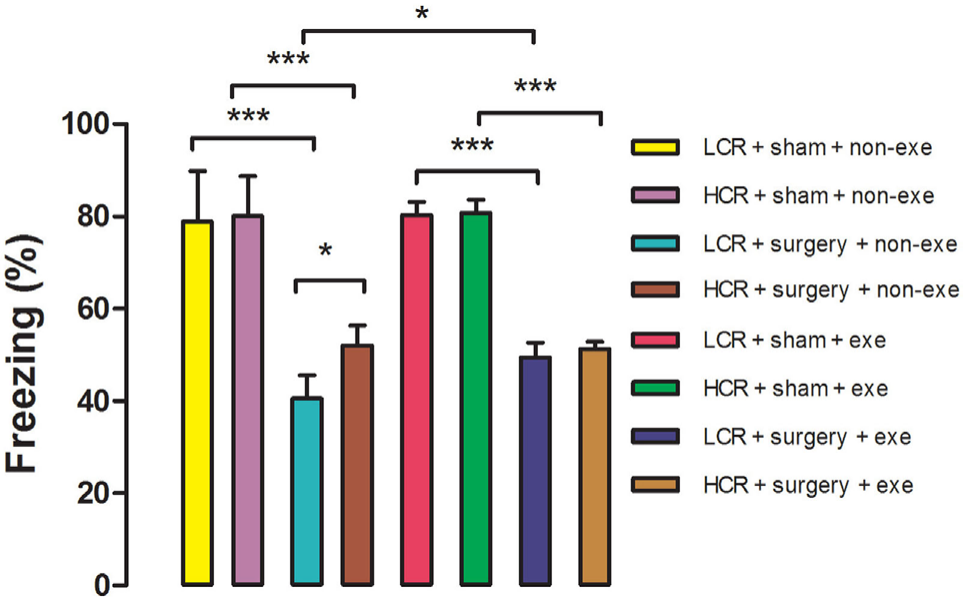
Preoperative exercise attenuated exaggerated acute cognitive decline in metabolic syndrome: 3 days after exercise (or no exercise) training, HCR and LCR rats (6–8/group) were subjected to preoperative training in a trace fear conditioning (TFC) paradigm. Surgery and sham surgery were performed with 30 min after training. Freezing behavior (represented as the as the % of time spent freezing over a 5 min observation period) was tested in the same TFC training context at 3 days after surgery. Percent time freezing is expressed as means ± SD, and analyzed by one-way ANOVA and Newman–Keuls *post hoc* analysis. *P < 0.05 and ***P < 0.001 for comparisons shown.

Acute postoperative memory, as assessed by percent of time spent freezing when returning to the same training context, was impaired in both HCR (80.1 ± 8.6 vs. 52 ± 4.3%, *P* < 0.001) and LCR (78.9 ± 10.9 vs. 40.5 ± 5.1%, *P* < 0.001) rats at 3 days after surgery (Figure 3). The decline in freezing time was greater in the LCR rats than the HCR rats (40.5 ± 5.1 vs. 52 ± 4.3%, *P* < 0.05), which is consistent with our previous report (11). Preoperative exercise rectified the exaggerated acute cognitive impairment in LCR rats (HCR + surgery + exercise vs. LCR + surgery + exercise, 51.3 ± 1.6 vs. 49.5 ± 3.1%, *P* > 0.05). Preoperative exercise in the LCR surgical group significantly improved the freezing time compared to the non-exercise LCR surgical group (49.5 ± 3.1 vs. 40.5 ± 5.1%, *P* < 0.05). Cohorts varied between 6 and 8/group.

### Preoperative Exercise Attenuated Persistent Cognitive Decline in Metabolic Syndrome Rats

**Figure 4:**
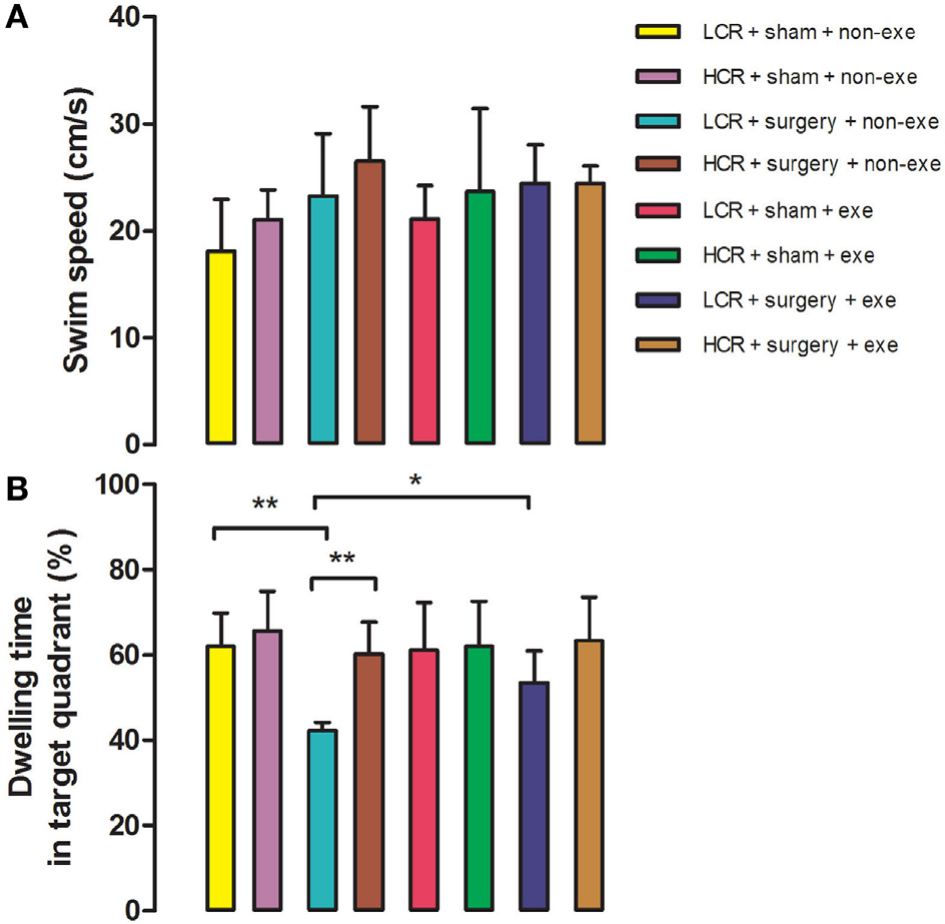
*Preoperative* exercise attenuated persistent cognitive decline in metabolic syndrome rats: 3 months after surgery, HCR and LCR rats were trained and tested in the Morris Water Maze paradigm. Swimming speed **(A)** and target quadrant dwelling time **(B)** as a percent of the total observation period of 60 s were recorded and expressed as mean ± SD, and analyzed by one-way ANOVA and Newman–Keuls *post hoc* analysis. **P < 0.01 for comparisons shown.

In the Morris Water Maze test, swimming speed was similar in all groups at 3 months after surgery (Figure 4). Long-term postoperative memory was assessed by dwelling time in the target quadrant in the probe trial of the Morris Water Maze test. The time that postoperative LCR rats spent in the quadrant in which the platform formerly resided was significantly shorter than for HCR rats (42.2 ± 1.9 vs. 60.2 ± 7.5%, *P* < 0.01), which is consistent with our previous report (11). Again, preoperative exercise rectified the persistent cognitive decline in LCR rats (HCR + surgery + exercise vs. LCR + surgery + exercise, 53.4 ± 7.5 vs. 63.3 ± 10.3%, *P* > 0.05). Preoperative exercise in the LCR surgical group significantly improved the dwell time compared to the non-exercise LCR surgical group (53.4 ± 7.5 vs. 42.2 ± 1.9% *P* < 0.05). Cohorts varied between 5 and 7/group.

### Preoperative Exercise Prevented Abnormal Neuroinflammation in Metabolic Syndrome Rats

**Figure 5:**
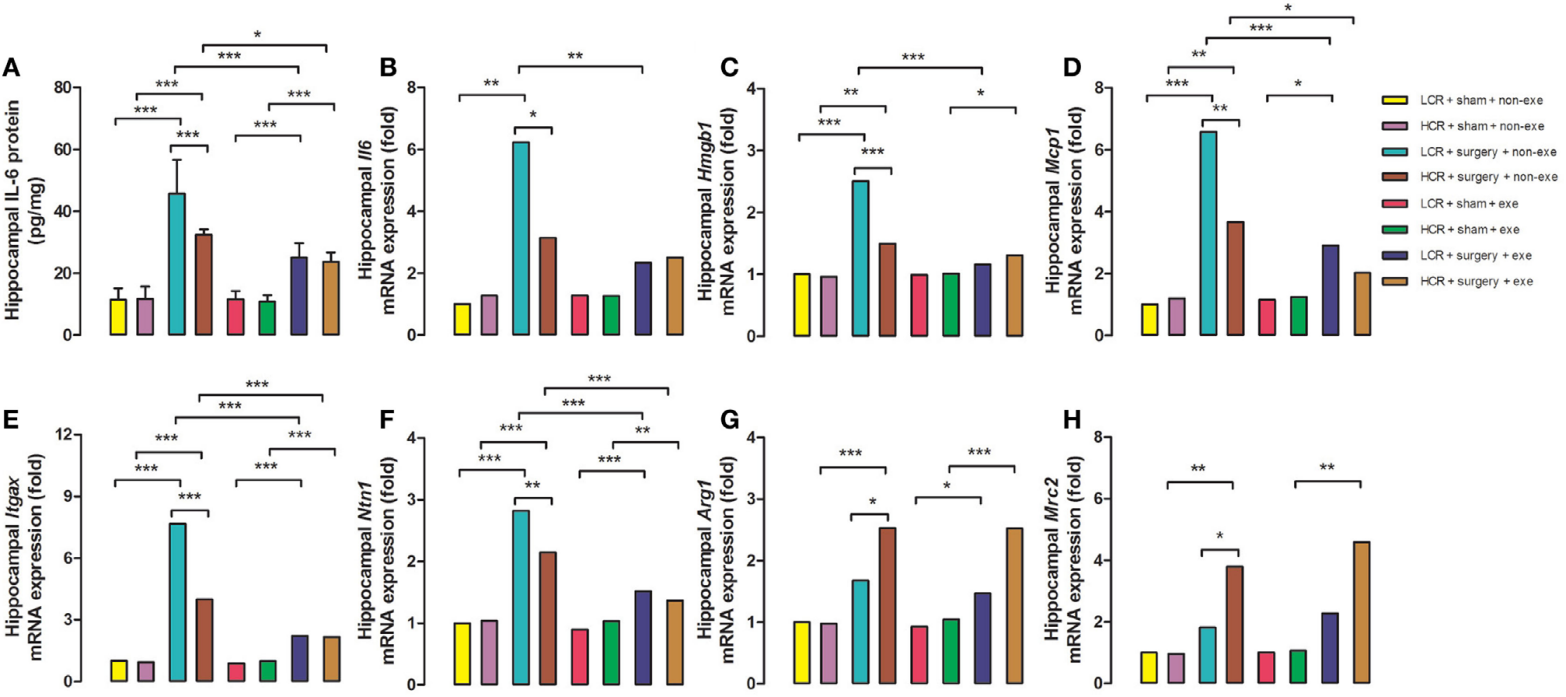
Preoperative exercise prevented abnormal neuroinflammation in metabolic syndrome rats: 3 days after surgery, the hippocampus was harvested from each group (*n* = 5–7/group). Protein expression of IL-6 **(A)** and mRNA expression of IL-6 **(B)**, HMGB-1 **(C)**, MCP-1 **(D)**, Itgax **(E)**, Netrin-1 **(F)**, Arg1 **(G)**, and Mrc2 **(H)** were measured. The data were expressed as means ± SD, and analyzed by one-way ANOVA and Newman–Keuls *post hoc* analysis. *P < 0.05, **P < 0.01, and ***P < 0.001 for comparisons shown.

Three days after surgery, hippocampal proinflammatory cytokine, IL-6 protein expression (Figure 5A), was remarkably elevated in LCR and HCR surgical rats with greater elevation in LCR surgical rats than HCR surgical rats (45.72 ± 10.88 vs. 32.41 ± 1.79 pg/mg, *P* < 0.001) (Figure 5). The mRNA levels for IL-6 (Figure 5B), HMGB1 (Figure 5C), MCP-1 (Figure 5D), Itgax (Figure 5E), and Netrin-1 (Figure 5F) were each increased in the surgical rats with LCR surgical rats exhibiting a significantly greater increase than the HCR surgical rats (6.2- vs. 3.1-fold increase for IL-6, *P* < 0.05; 2.5- vs. 1.5-fold increase for HMGB-1, *P* < 0.001; 6.6- vs. 3.7-fold increase for MCP-1, *P* < 0.01; 7.7- vs. 4-fold increase for Itgax, *P* < 0.001; 2.8- vs. 2.1-fold increase for Netrin-1, *P* < 0.01). The mRNA levels of the pro-resolving mediators Arg1 (Figure 5G) and Mrc2 (Figure 5H) were significantly increased in both HCR and LCR rats although the increase in HCR rats was greater than in the LCR rats (2.5- vs. 1.7-fold increase for Arg1, *P* < 0.05; 3.8- vs. 1.8-fold increase for Mrc2, *P* < 0.05). For each of the genes, preoperative exercise eliminated the differences in the surgery-induced changes in the proinflammatory (Figures 5A–F) mediators. Exercise significantly attenuated the increase in pro-inflammatory cytokines in the postoperative LCR rats (IL-6 protein expression 24.97 ± 4.76 vs. 45.72 ± 10.88 pg/mg, *P* < 0.001; IL-6 mRNA 2.3- vs. 6.2-fold increase, *P* < 0.01; HMGB-1 mRNA 1.2- vs. 2.5-fold, *P* < 0.001; MCP-1 mRNA 2.9- vs. 6.6-fold, *P* < 0.001; Itgax mRNA 2.2- vs. 7.7-fold, *P* < 0.001; Netrin-1 mRNA 1.5- vs. 2.8-fold, *P* < 0.001). Cohorts varied between 5 and 7/group.

### Preoperative Exercise Rectified Abnormal Microbiome LCR Rats

**Figure 6:**
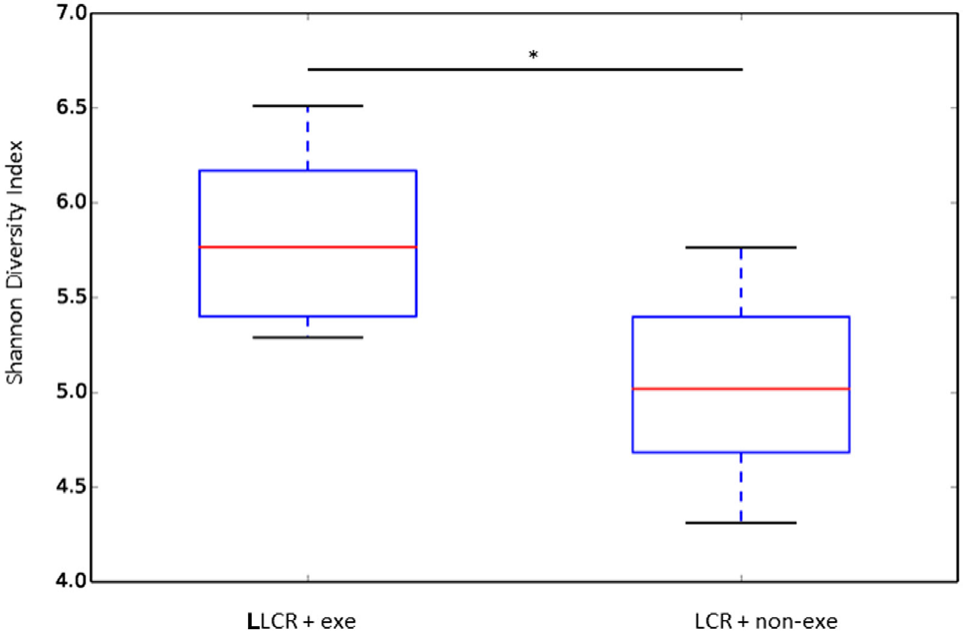
Exercise rectified α diversity of the fecal microbiome in metabolic syndrome rats: low capacity runner rats were divided into exercise and non-exercise groups (*n* = 6) and stools were collected after 6 weeks of exercise or no exercise for microbiome assessment. The α diversity of the fecal microbiome was calculated by the Shannon method. Data are expressed as box and whisker plots with median flanked by first and third quartiles and variance bars showing the extremes. Groups were compared by *t*-test. *P < 0.03.

The α diversity of the gut microbiome in the LCR rats (*n* = 6/group) was significantly improved after exercise (Figure 6) while no change was noted after exercise in the HCR rats (data not shown) (Figure 6). Exercise significantly improved the β diversity in both the LCR and HCR groups and significantly altered the abundance of two of the major phyla, Firmicutes and Bacteroidetes in the HCR rats (See Supplementary Material).

## Discussion

### Major Findings

In LCR rats, preoperative exercise attenuated both the exaggerated acute postoperative decrease in freezing time at day 3 (Figure 3) as well as the decrement in recall (dwelling time) in the Morris Water Maze probe trial performed three months postoperatively (Figure 4). The enhanced neuroinflammatory response to surgical trauma was attenuated by exercise in the LCR rats (Figures 5A–F). The α diversity of the gut microbiome was significantly improved by exercise in the LCR rats (Figure 6). In aggregate these data suggest that preoperative exercise can rectify the neuroinflammatory response to surgery resulting in an elimination of the exaggerated and persistent PCD. The exercise-induced improvement in postoperative neuroinflammation and cognition in the LCR rats was associated with a significant improvement in gut dysbiosis.

### Metabolic Syndrome, Inflammation, Effects of Exercise

Over the past two decades Britton and Koch produced two cohorts of rats that, at generation 35, differ more than 10-fold in their treadmill running capacity at peak exhaustion (21). The rapid fatigability contributes to the sedentary behavior of the LCR rats (22) that is the likely antecedent (23) to their development of obesity, hypertension, hyperlipidemia, and insulin resistance (24). Earlier, we had demonstrated that the LCR rats exhibit a more exaggerated and persistent cognitive decline (11) that is associated with failure of both the neural and humoral inflammation-resolution pathways (20). The hyperinflammatory states that are observed in obesity and insulin resistance have been termed “metainflammation” which is thought to contribute to the comorbidities that occur in these metabolically deranged states (25). The failure to appropriately resolve inflammation in the LCR rats results in a relative increased neuroinflammatory response (Figures 5A–F) and a relative decrease in the pro-resolving response (Figures 5G,H) when compared to the HCR rats. These altered postoperative neuroinflammatory responses were corrected by preoperative exercise in the LCR rats (Figures 5A–H).

### Gut Microbiome, Obesity, Inflammation, and Exercise

Characteristics of a healthy microbiome include community stability and increased species diversity. Obesity is associated with reduced bacterial diversity in gut microbiome (26). Similarly, a lower level of bacterial diversity was also observed in the setting of the metabolic syndrome (27) and the altered microbiome may contribute to hyperlipidemia (28). As the gut microbiome can perform functions that modulate the immune system (29) it is unsurprising that changes in the microbiome can influence the inflammatory response. The gut microbiome is in a state of dynamic homeostasis with the immune and nervous systems (30, 31). In a mouse model of Alzheimer’s disease, change in peripheral cytokines and microglia occurred pari passu with alteration of the gut microbiome induced by broad-spectrum oral antibiotics (32). With advanced age there is less diversity in the gut microbiome (33) and the chronic low-grade inflammatory response associated with aging, referred to as “inflammaging,” has recently been linked to age-related microbiome changes (34).

Several recent mice studies have now shown a direct link between exercise and beneficial changes in the gut microbiome. The dysbiosis present in high fat diet-induced obese mice was corrected by exercise (35). In another high-fat diet mouse study, exercise did induce microbiota changes but these alterations were not the same as those produced by the high-fat diet (36).

In the current study there was a significant improvement in the α (Figure 6) and the β diversity (see Figure S1 in Supplementary Material) of the gut microbiome following exercise in the LCR rats. An α diversity index (such as the Shannon index) reflects the species diversity in a community and takes into account not only species richness but also the relative abundances of different species (evenness). Thus, the α diversity index indicates how many types and how equally the microbiome is present in one subject. The β diversity represents a difference in species diversity between different environments and illustrates exchange or similarity of species between compared environments.

Our data, which demonstrate that an exercise-induced improvement in the diversity of the gut microbiome of the LCR rats (Figure 6) is associated with a normalization of the neuroinflammatory response (Figure 5), are corroborated by previous reports that a less diverse gut microbiome is associated with a hyperinflammatory state (34). Interestingly, the exercise-induced changes in the taxonomic profiles that were observed in both the LCR and HCR rats involved an increase Firmicutes and a decrease in Bacteroidetes although the change was greater and statistically significant for the HCR rats (see Figure S2 in Supplementary Material). Re-assuringly, the direction of these exercise-induced changes in the taxonomic profiles of these two major phyla was similar to that observed in post-exercise high-fat diet obese mice (36). The sequence data are provided in Table S1 in Supplementary Material.

### Caveats

Although we had observed exercise-induced changes in the gut microbiome that were associated with less postoperative neuroinflammation and cognitive decline, it is not possible to ascribe a cause and effect relationship between the improvement in the diversity of the gut microbiome and the attenuation of the surgical phenotype. In order to address the causal nature of the abnormal microbiome in the LCR rats for the exaggerated and persistent surgical phenotype, future studies will need to address the direct effects of altering the microbiome, possibly by diet or fecal transplantation.

### Implications

PCD imposes significant costs on patients, their families and caretakers, and more broadly on society as a whole. A recent meta-analysis revealed that in older patients, delirium is associated with an increased risk of death, institutionalization, and dementia (37). Understanding the postoperative pathophysiological mechanisms and developing an intervention with the potential to reduce PCD have the potential for significant clinical impact. Of course, not all patients awaiting surgery will be able to exercise, either because of their physical condition or because the surgery is urgent. However, even these patients may benefit from the knowledge of the changes that exercise induces in the innate immune system as these could be a target for a different type of intervention designed, for example, to enhance the diversity and stability of the gut microbiome.

## Ethics Statement

All experimental procedures involving animals were approved by Institutional Animal Care and Use Committee (IACUC) of University of California, San Francisco (protocol no.: AN167062), and conformed to National Institute of Health guidelines.

## Author Contributions

XF and YU contributed equally as first authors. XF performed the behavioral studies that were performed at 3 days and 3 months and contributed to the writing of the manuscript. YU collected samples, analyzed samples, and participated in the microbiome analysis and contributed to the writing of the manuscript. LK generated the LCR and HCR rats. SB generated the LCR and HCR rats. JH performed exercise studies. DL performed the exercise studies. MM conceived of the experiment, obtained the funding, and wrote the manuscript.

## Conflict of Interest Statement

The authors declare that the research was conducted in the absence of any commercial or financial relationships that could be construed as a potential conflict of interest.

## References

[1] Avidan MS Evers AS The fallacy of persistent postoperative cognitive decline. Anesthesiology (2016) 124(2):255–8. 10.1097/ALN.0000000000000958 26785428 PMC5839806

[2] Vacas S Degos V Tracey KJ Maze M. High-mobility group box 1 protein initiates postoperative cognitive decline by engaging bone marrow-derived macrophages. Anesthesiology (2014) 120(5):1160–7. 10.1097/ALN.0000000000000045 24162463 PMC3999217

[3] Terrando N Eriksson LI Ryu JK Yang T Monaco C Feldmann M Resolving postoperative neuroinflammation and cognitive decline. Ann Neurol (2011) 70(6):986–95. 10.1002/ana.22664 22190370 PMC4556354

[4] Feng X Valdearcos M Uchida Y Lutrin D Maze M Koliwad SK. Microglia mediate postoperative hippocampal inflammation and cognitive decline in mice. JCI Insight (2017) 2(7):e91229. 10.1172/jci.insight.91229 28405620 PMC5374063

[5] Terrando N Gomez-Galan M Yang T Carlstrom M Gustavsson D Harding RE Aspirin-triggered resolvin D1 prevents surgery-induced cognitive decline. FASEB J (2013) 27(9):3564–71. 10.1096/fj.13-230276 23709617

[6] Pavlov VA Tracey KJ. Neural regulation of immunity: molecular mechanisms and clinical translation. Nat Neurosci (2017) 20(2):156–66. 10.1038/nn.4477 28092663

[7] Serhan CN. Discovery of specialized pro-resolving mediators marks the dawn of resolution physiology and pharmacology. Mol Aspects Med (2017) 58:1–11. 10.1016/j.mam.2017.03.001 28263773 PMC5582020

[8] Hudetz JA Patterson KM Iqbal Z Gandhi SD Pagel PS. Metabolic syndrome exacerbates short-term postoperative cognitive dysfunction in patients undergoing cardiac surgery: results of a pilot study. J Cardiothorac Vasc Anesth (2011) 25(2):282–7. 10.1053/j.jvca.2010.06.008 20728380

[9] Hudetz JA Patterson KM Amole O Riley AV Pagel PS. Postoperative cognitive dysfunction after noncardiac surgery: effects of metabolic syndrome. J Anesth (2011) 25(3):337–44. 10.1007/s00540-011-1137-0 21516370

[10] Koch LG Britton SL. Divergent selection for aerobic capacity in rats as a model for complex disease. Integr Comp Biol (2005) 45(3):405–15. 10.1093/icb/45.3.405 21676786

[11] Feng X Degos V Koch LG Britton SL Zhu Y Vacas S Surgery results in exaggerated and persistent cognitive decline in a rat model of the Metabolic Syndrome. Anesthesiology (2013) 118(5):1098–105. 10.1097/ALN.0b013e318286d0c9 23353794 PMC5530762

[12] Sloan RP Shapiro PA Demeersman RE McKinley PS Tracey KJ Slavov I Aerobic exercise attenuates inducible TNF production in humans. J Appl Physiol (1985) (2007) 103(3):1007–11. 10.1152/japplphysiol.00147.2007 17626836

[13] Gleeson M Bishop NC Stensel DJ Lindley MR Mastana SS Nimmo MA. The anti-inflammatory effects of exercise: mechanisms and implications for the prevention and treatment of disease. Nat Rev Immunol (2011) 11(9):607–15. 10.1038/nri3041 21818123

[14] You T Arsenis NC Disanzo BL Lamonte MJ Effects of exercise training on chronic inflammation in obesity: current evidence and potential mechanisms. Sports Med (2013) 43(4):243–56. 10.1007/s40279-013-0023-3 23494259

[15] Olivo CR Miyaji EN Oliveira ML Almeida FM Lourenco JD Abreu RM Aerobic exercise attenuates pulmonary inflammation induced by *Streptococcus pneumoniae*. J Appl Physiol (1985) (2014) 117(9):998–1007. 10.1152/japplphysiol.00290.2014 25190745

[16] Beiter T Hoene M Prenzler F Mooren FC Steinacker JM Weigert C Exercise, skeletal muscle and inflammation: ARE-binding proteins as key regulators in inflammatory and adaptive networks. Exerc Immunol Rev (2015) 21:42–57. 25826388

[17] Annibalini G Lucertini F Agostini D Vallorani L Gioacchini A Barbieri E Concurrent aerobic and resistance training has anti-inflammatory effects and increases both plasma and leukocyte levels of IGF-1 in late middle-aged type 2 diabetic patients. Oxid Med Cell Longev (2017) 2017:3937842. 10.1155/2017/3937842 28713486 PMC5497609

[18] Lynch SV Pedersen O The human intestinal microbiome in health and disease. N Engl J Med (2016) 375(24):2369–79. 10.1056/NEJMra1600266 27974040

[19] Koch LG Britton SL. Artificial selection for intrinsic aerobic endurance running capacity in rats. Physiol Genomics (2001) 5(1):45–52. 11161005 10.1152/physiolgenomics.2001.5.1.45

[20] Su X Feng X Terrando N Yan Y Chawla A Koch LG Dysfunction of inflammation-resolving pathways is associated with exaggerated postoperative cognitive decline in a rat model of the metabolic syndrome. Mol Med (2013) 18:1481–90. 10.2119/molmed.2012.00351 23296426 PMC3576477

[21] Garton FC North KN Koch LG Britton SL Nogales-Gadea G Lucia A. Rodent models for resolving extremes of exercise and health. Physiol Genomics (2016) 48(2):82–92. 10.1152/physiolgenomics.00077.2015 26395598 PMC4729696

[22] Novak CM Escande C Burghardt PR Zhang M Barbosa MT Chini EN Spontaneous activity, economy of activity, and resistance to diet-induced obesity in rats bred for high intrinsic aerobic capacity. Horm Behav (2010) 58(3):355–67. 10.1016/j.yhbeh.2010.03.013 20350549 PMC2923555

[23] Sisson SB Camhi SM Church TS Martin CK Tudor-Locke C Bouchard C Leisure time sedentary behavior, occupational/domestic physical activity, and metabolic syndrome in U.S. men and women. Metab Syndr Relat Disord (2009) 7(6):529–36. 10.1089/met.2009.0023 19900152 PMC2796695

[24] Noland RC Thyfault JP Henes ST Whitfield BR Woodlief TL Evans JR Artificial selection for high-capacity endurance running is protective against high-fat diet-induced insulin resistance. Am J Physiol Endocrinol Metab (2007) 293(1):E31–41. 10.1152/ajpendo.00500.2006 17341547

[25] Singer K Lumeng CN. The initiation of metabolic inflammation in childhood obesity. J Clin Invest (2017) 127(1):65–73. 10.1172/JCI88882 28045405 PMC5199687

[26] Turnbaugh PJ Hamady M Yatsunenko T Cantarel BL Duncan A Ley RE A core gut microbiome in obese and lean twins. Nature (2009) 457(7228):480–4. 10.1038/nature07540 19043404 PMC2677729

[27] Lim MY You HJ Yoon HS Kwon B Lee JY Lee S The effect of heritability and host genetics on the gut microbiota and metabolic syndrome. Gut (2017) 66(6):1031–8. 10.1136/gutjnl-2015-311326 27053630

[28] Fu J Bonder MJ Cenit MC Tigchelaar EF Maatman A Dekens JA The gut microbiome contributes to a substantial proportion of the variation in blood lipids. Circ Res (2015) 117(9):817–24. 10.1161/CIRCRESAHA.115.306807 26358192 PMC4596485

[29] Eid HM Wright ML Anil Kumar NV Qawasmeh A Hassan STS Mocan A Significance of microbiota in obesity and metabolic diseases and the modulatory potential by medicinal plant and food ingredients. Front Pharmacol (2017) 8:387. 10.3389/fphar.2017.00387 28713266 PMC5493053

[30] Belkaid Y Harrison OJ. Homeostatic immunity and the microbiota. Immunity (2017) 46(4):562–76. 10.1016/j.immuni.2017.04.008 28423337 PMC5604871

[31] Rea K Dinan TG Cryan JF. The microbiome: a key regulator of stress and neuroinflammation. Neurobiol Stress (2016) 4:23–33. 10.1016/j.ynstr.2016.03.001 27981187 PMC5146205

[32] Minter MR Zhang C Leone V Ringus DL Zhang X Oyler-Castrillo P Antibiotic-induced perturbations in gut microbial diversity influences neuro-inflammation and amyloidosis in a murine model of Alzheimer’s disease. Sci Rep (2016) 6:30028. 10.1038/srep30028 27443609 PMC4956742

[33] Biagi E Nylund L Candela M Ostan R Bucci L Pini E Through ageing, and beyond: gut microbiota and inflammatory status in seniors and centenarians. PLoS One (2010) 5(5):e10667. 10.1371/journal.pone.0010667 20498852 PMC2871786

[34] Buford TW. (Dis)Trust your gut: the gut microbiome in age-related inflammation, health, and disease. Microbiome (2017) 5(1):80. 10.1186/s40168-017-0296-0 28709450 PMC5512975

[35] Campbell SC Wisniewski PJ Noji M McGuinness LR Haggblom MM Lightfoot SA The effect of diet and exercise on intestinal integrity and microbial diversity in mice. PLoS One (2016) 11(3):e0150502. 10.1371/journal.pone.0150502 26954359 PMC4783017

[36] Evans CC LePard KJ Kwak JW Stancukas MC Laskowski S Dougherty J Exercise prevents weight gain and alters the gut microbiota in a mouse model of high fat diet-induced obesity. PLoS One (2014) 9(3):e92193. 10.1371/journal.pone.0092193 24670791 PMC3966766

[37] Witlox J Eurelings LS de Jonghe JF Kalisvaart KJ Eikelenboom P van Gool WA. Delirium in elderly patients and the risk of postdischarge mortality, institutionalization, and dementia: a meta-analysis. JAMA (2010) 304(4):443–51. 10.1001/jama.2010.1013 20664045

