# Supplementary material for: Exercise Prevents Enhanced Postoperative Neuroinflammation and Cognitive Decline and Rectifies the Gut Microbiome in a Rat Model of Metabolic Syndrome
Source: Front Immunol. 2017 Dec 11;8:1768. doi: 10.3389/fimmu.2017.01768 (PMC5732173; doi:10.3389/fimmu.2017.01768)
Supplement: Supplementary file 1 [file Data_Sheet_1.docx]

**Supplemental Information**

Methods

*Assessment of β Diversity of the Fecal Microbiome*

As a downstream analysis from the rarefied OTU table, Unweighted UniFrac dissimilarity matrices were generated in QIIME software to compare the similarity of the microbiome compositions between different environments. Principal Coordinate Analysis (PCoA) plots were used to visualize multivariate data as a low dimensional spatial arrangement. Permutational multivariate analysis of variance (PERMANOVA) was used to determine significance in dissimilarity matrices.

*Assessment of Major Phyla of the Fecal Microbiome*

The abundance of the two major bacterial phyla, established by rarefied sequencing reads, were compared among LCR and HCR rats before and after exercise.

Results

The β diversity was significantly altered by exercise in both the LCR and HCR rats (Supplemental Figure 1).

After exercise there was a significant decrease of Bacteroidetes and a significant increase in Firmicutes only in the HCR rats (Supplemental Figure 2).

The RNA Sequencing data are accessible from the National Center for Biotechnology Information (NCBI) (<https://www.ncbi.nlm.nih.gov/Traces/study/?acc=SRP125513>) as listed in Supplementary Table 1.

Supplemental Table 1: Features of the RNA Sequencing Data Deposited in NCBI

Legends

Supplemental Figure 1: *Exercise rectified β diversity of the fecal microbiome in LCR and HCR rats*

Rats were divided into exercise and non-exercise groups (n=6) and stools were collected after 6 weeks of exercise or no exercise for β diversity assessment by the Unweighted UniFrac method.

Supplemental Figure 1: *Effect of exercise on abundance of Bacteroidetes and Firmicutes in LCR and HCR rats*

Rats were divided into exercise and non-exercise groups (n=6) and stools were collected after 6 weeks of exercise (exe) or no exercise (non-exe) for assessment of the major phyla. * =P<0.05
