# Supplementary figures and images for: Exercise Prevents Enhanced Postoperative Neuroinflammation and Cognitive Decline and Rectifies the Gut Microbiome in a Rat Model of Metabolic Syndrome
Source: Front Immunol. 2017 Dec 11;8:1768. doi: 10.3389/fimmu.2017.01768 (PMC5732173; doi:10.3389/fimmu.2017.01768)

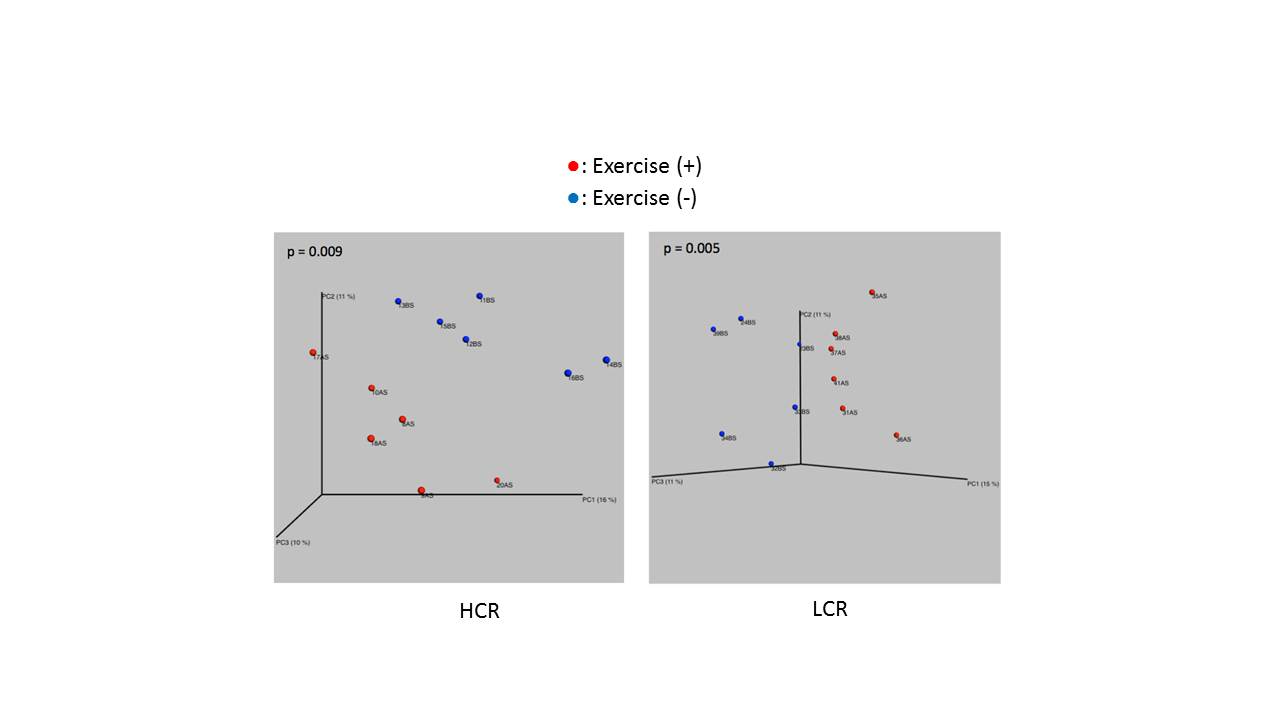

Supplement: Supplementary file 2 [file Image_1.tif]

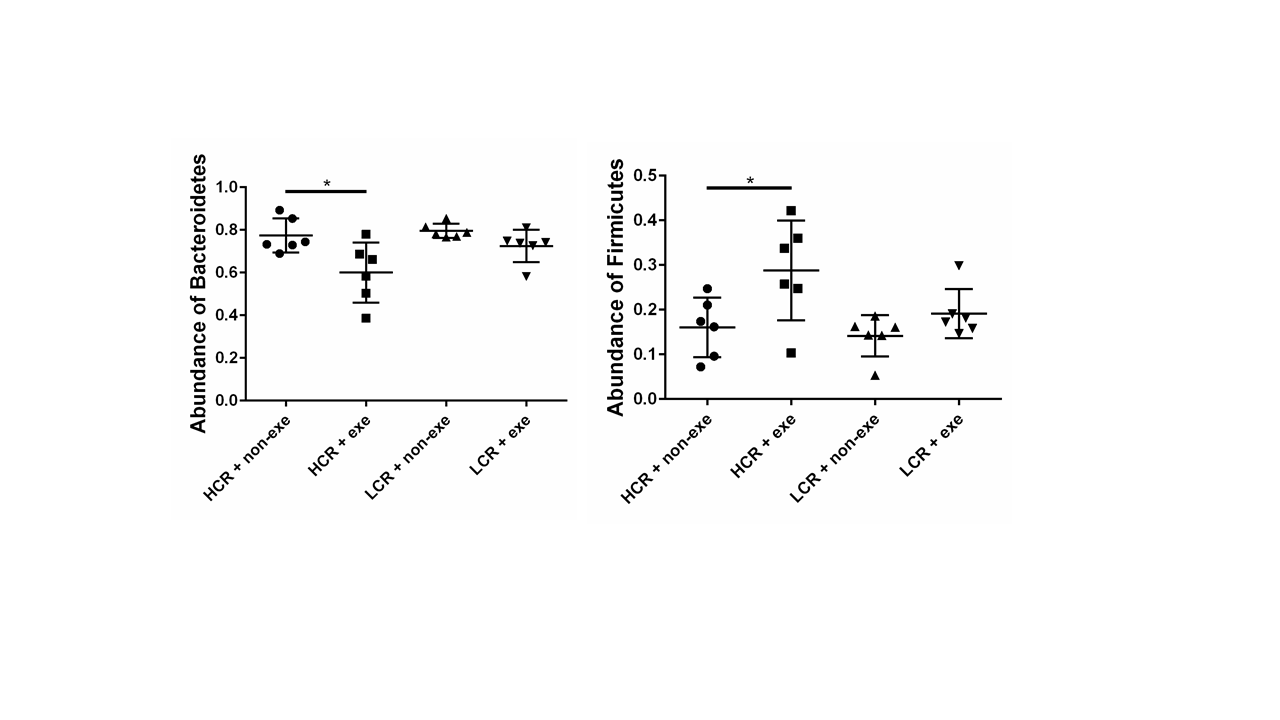

Supplement: Supplementary file 3 [file Image_2.tif]
